# Knowledge of Non-Vitamin K Antagonist Oral Anticoagulants and Associated Factors Among Patients with Atrial Fibrillation: A Cross-Sectional Study in Thailand

**DOI:** 10.3390/healthcare14182979

**Published:** 2026-09-12

**Authors:** Siriporn Jantharuechai, Pairot Khusakul, Wannee Chaichalermpong, Chaiyasith Wongvipaporn, Verawan Uchaipichat

**Affiliations:** 1 Pharmacy Department, Queen Sirikit Heart Center of the Northeast, Faculty of Medicine, Khon Kaen University, Khon Kaen 40002, Thailand; sirijan@kku.ac.th; 2 Division of Clinical Pharmacy, Faculty of Pharmaceutical Sciences, Khon Kaen University, Khon Kaen 40002, Thailand; 3 Division of Social and Administration, Faculty of Pharmaceutical Sciences, Khon Kaen University, Khon Kaen 40002, Thailand; wancha2@kku.ac.th; 4 Cardiovascular Unit, Department of Medicine, Faculty of Medicine, Khon Kaen University, Khon Kaen 40002, Thailand; chawon@kku.ac.th

**Keywords:** anticoagulant, atrial fibrillation, NOACs, patient medication knowledge, Thailand

## Abstract

**Highlights:**

**What are the main findings?**

- Despite the majority of Thai AF patients receiving NOACs demonstrating high knowledge, critical gaps were identified in laboratory monitoring awareness and dose management in terms of missed dose, double dosing, and medication effectiveness after vomiting.
- Older age (≥75 years), education below tertiary level, and lack of awareness of NOAC indications were independently associated with low NOAC knowledge.

**What are the implications of the main findings?**

- Targeted educational interventions should prioritise elderly patients, those with lower educational attainment, and those who are unaware of their treatment indication—patient groups consistently identified as at higher risk of low anticoagulation knowledge across diverse settings.
- Structured patient education addressing laboratory monitoring requirements and dose management is essential to optimise NOAC therapy safety and improve clinical outcomes in AF patients.

**Abstract:**

**Background/Objectives:** Adequate patient knowledge of non-vitamin K antagonist oral anticoagulants (NOACs) is important for their safe and effective use in atrial fibrillation (AF), yet knowledge deficits remain widely reported. This study assessed NOAC knowledge and identified factors associated with knowledge level among Thai AF outpatients at a tertiary cardiac center. **Methods:** A cross-sectional study was conducted between January and March 2023 using the KAQNAF-Thai, a validated 23-item instrument covering six domains, classified as high (≥60%) or low (<60%). Factors associated with low knowledge were identified using multivariable logistic regression. **Results:** A total of 250 participants were enrolled (mean age 71.9 ± 9.13 years; 53.2% male). The mean total knowledge score was 15.4 ± 3.04 out of 23 points (67% correct responses), with 178 (71.2%) having high knowledge and 72 (28.8%) having low knowledge. The most critical gaps were in laboratory monitoring (correct response rate 36.0%) and dose management; accidental double dosing (13.2%), NOAC effectiveness after vomiting (30.4%), and missed dose management (34.4%). Three variables were independently associated with low knowledge: age ≥75 years (aOR = 2.11 [95% CI 1.10–4.05]), education below tertiary level (aOR = 17.38 [95% CI 5.03–60.01]), and lack of awareness of NOAC indications (aOR = 3.22 [95% CI 1.25–8.29]). **Conclusions:** Although most Thai AF patients met the threshold for high NOAC knowledge, critical gaps in laboratory monitoring and dose management persisted even in this group. Educational interventions should prioritise elderly patients, those with lower educational attainment, and those unaware of their NOAC indication.

## 1. Introduction

Atrial fibrillation (AF) is the most common type of sustained cardiac arrhythmia in adults and is associated with a fivefold increase in stroke risk, increased mortality, and impaired quality of life [1,2]. Its prevalence increases markedly with age and continues to increase globally, including in Thailand [3,4]. Stroke prevention through oral anticoagulation is therefore a cornerstone of AF management. Vitamin K antagonists (VKAs), such as warfarin, have served as the mainstay of therapy for over five decades; however, their narrow therapeutic index, unpredictable pharmacodynamic responses, susceptibility to drug interactions, and requirement for regular INR monitoring present substantial practical challenges [2,5]. These limitations drove the development of non-vitamin K antagonist oral anticoagulants (NOACs)—comprising dabigatran, rivaroxaban, apixaban, and edoxaban—which offer fixed dosing, more predictable anticoagulant effects, fewer drug interactions, and no requirement for routine coagulation monitoring [6]. Large phase 3 clinical trials have demonstrated that NOACs are non-inferior or superior to warfarin for stroke prevention with a more favourable bleeding profile, and current international guidelines recommend NOACs as the preferred anticoagulant in eligible patients [2,7].

Unlike VKAs, which act indirectly, NOACs directly inhibit either factor Xa or thrombin, producing rapid and predictable anticoagulant effects [6]. Consequently, even a single missed dose can meaningfully reduce anticoagulant protection, making adherence a critical determinant of therapeutic outcomes [8,9]. Patient knowledge of anticoagulant therapy has been consistently identified as a key determinant of adherence and clinical outcomes in studies of VKA users [10,11,12], and this relationship has similarly been reported in studies involving mixed populations of VKA and NOAC users [13,14,15]. Indeed, the European Heart Rhythm Association (EHRA) practical guide on NOAC use explicitly recommends that patient education regarding the benefits and risks of anticoagulation, the importance of adherence, and the specific characteristics of NOACs should be provided at treatment initiation and reinforced at every follow-up visit [16]. Nevertheless, NOAC knowledge among AF patients has been found to be generally low to moderate across multiple settings [14,17,18,19]. Interestingly, these deficits are consistently more pronounced among patients taking NOACs than among those taking VKAs [17,18]. Obamiro et al. and Metaxas et al. suggested that this disparity may reflect the more frequent healthcare provider contact required for VKA monitoring, which could provide greater opportunity for patient education than the simplified monitoring schedule typical of NOAC therapy; however, this explanation was not directly tested in either study and warrants further investigation [18,20]. This disparity is of particular clinical concern given that NOACs are now the dominant anticoagulant prescribed in AF.

Despite the growing use of NOACs in clinical practice, tools and studies assessing anticoagulation knowledge in Thailand have focused predominantly on warfarin users [21,22,23]. Although validated NOAC knowledge instruments exist internationally, psychometric properties are specific to the population in which an instrument was developed and cannot be assumed to be directly applicable elsewhere without translation and re-validation, given differences in language, health literacy, and healthcare-system context. To address this gap, our research group recently developed and validated the Knowledge Assessment Questionnaire on NOAC Use in Thai Patients with Atrial Fibrillation (KAQNAF-Thai) at the Queen Sirikit Heart Center of the Northeast, a tertiary cardiac referral center in northeastern Thailand [24]. However, the application of this tool to assess NOAC knowledge and identify its associated factors in an independent sample of Thai AF patients has not yet been undertaken.

This study aimed to apply the KAQNAF-Thai to assess the level of NOAC knowledge among AF outpatients receiving NOACs at the Queen Sirikit Heart Center of the Northeast, and to examine the cross-sectional association between NOAC knowledge level and pre-specified demographic and clinical factors, namely age, sex, education level, duration of NOAC use, presence of comorbidities, number of concomitant medications, and awareness of NOAC indications.

## 2. Materials and Methods

### 2.1. Study Design and Setting

A cross-sectional study was conducted at the outpatient clinic of the Queen Sirikit Heart Center of the Northeast, Khon Kaen University, Thailand.

### 2.2. Participants

Eligible participants were AF outpatients receiving NOACs at the Queen Sirikit Heart Center of the Northeast who met the following criteria: aged 18 years or older; were diagnosed with atrial fibrillation; received NOACs, including rivaroxaban, dabigatran, apixaban, or edoxaban, for a minimum of three months; were capable of self-administering their medication; and were able to read and write in Thai. A convenience sampling approach was used, whereby eligible patients attending the outpatient pharmacy for routine dispensing during the study period were approached and screened against these criteria until the target sample size was reached. Patients who had participated in the original development and validation study of the KAQNAF-Thai were excluded, ensuring that the present study sample was entirely independent of the instrument validation cohort. Patients who declined to participate were excluded. A prospective screening log was not maintained; therefore, the exact numbers of patients approached, found ineligible, or declining participation could not be recorded.

### 2.3. Data Collection

Participants were recruited at the outpatient pharmacy during routine visits between 14 January and 17 March 2023. After providing written informed consent, data were collected using a structured, self-administered questionnaire completed in a private counselling area. The questionnaire consisted of two sections: (i) demographic characteristics and potential factors associated with NOAC knowledge level, including age, sex, education level, duration of NOAC therapy, presence of comorbidities and number of concomitant medications, and (ii) the KAQNAF-Thai, a validated instrument developed by our research group [24]. The original questionnaire was administered in Thai, and an English translation was provided for the purpose of this publication (Supplementary S1).

### 2.4. Evaluation Criteria for Knowledge Assessment

The KAQNAF-Thai comprises 23 items across six domains (Supplementary S1), presented in a three-choice format of true/false/”not sure” or content-specific multiple-choice options. Items 1.1 and 3.1, which lack a fixed answer key as they are scored against the participant’s prescribed regimen, were administered verbally by a single research pharmacist to eliminate inter-interviewer variability; each item was read verbatim, with no prompting, rewording, or confirmatory feedback provided, and responses were recorded and subsequently verified against the dispensing record. One point was awarded for each correct answer, and zero points were awarded for incorrect or “not sure” responses, yielding a maximum possible score of 23. The knowledge level was classified as high (score ≥14, ≥60%) or low (score <14, <60%). The 60% threshold was modified from Bloom’s cut-off criteria, which distinguishes poor knowledge (<60%) from moderate (60–79%) and good (≥80%). As in Li et al., who applied a comparable dichotomised cutoff for multivariable logistic regression in a similar AF population [14], we combined the moderate and good categories into a single ‘high knowledge’ group to maintain statistical power given our sample size. The total score is also reported as median (IQR) alongside the mean ± SD to better characterise its distribution.

### 2.5. Sample Size

The sample size was estimated using the events per variable (EPV) criterion, which requires at least 10 participants from the smaller outcome group per independent variable [25]. Seven independent variables—age, sex, education level, duration of NOAC use, presence of comorbidities, number of concomitant medications, and awareness of NOAC indications—were pre-specified based on factors previously reported to be associated with anticoagulation knowledge in the literature [11,13,14,18,19,20]. A formal logistic regression power calculation, which requires pre-specification of an expected effect size, significance level, and power, was not feasible, as no prior Thai data existed on NOAC knowledge to inform an anticipated effect size for any of these seven predictors. In the absence of local prevalence data, an equal distribution of knowledge levels (50% each) was conservatively assumed, as this yields the largest and therefore most conservative required sample size regardless of the true underlying distribution. Based on these assumptions, a minimum of 140 participants was required. After a 20% adjustment for potential non-response, the minimum was 168 participants. A final sample size of 250 participants was used, which was feasible within the study period.

### 2.6. Statistical Analysis

Descriptive statistics were used to summarise participant characteristics, with continuous variables presented as means ± SD or median (interquartile range, IQR) as appropriate, and categorical variables presented as frequencies and percentages. Univariate analysis was performed using Pearson’s chi-square or Fisher’s exact tests, with variables meeting a *p* value < 0.20 threshold entered into a multivariable logistic regression model to identify factors associated with low knowledge level. Although no single *p*-value threshold for univariate screening is universally standardised in the literature, the 0.20 threshold used in this study is a relatively conservative choice and is more lenient than—and therefore less likely to exclude potentially important variables than—the thresholds applied in comparable studies of anticoagulation knowledge, which used *p* < 0.05 [14] and *p* < 0.10 [18]. Adjusted odds ratios (aORs) with 95% confidence intervals are reported. Multicollinearity was assessed using tolerance and variance inflation factor (VIF) values, and influential observations were evaluated using Cook’s distance, leverage values, and DFBETA statistics. Model discrimination was assessed using the area under the receiver operating characteristic curve (AUC), and calibration was evaluated using the Hosmer–Lemeshow goodness-of-fit test and by comparing predicted probabilities with observed outcomes across deciles of predicted probability. The number of events per model parameter was also calculated. Statistical significance was set at *p* value < 0.05, and all tests were two-tailed. Analyses were performed using IBM SPSS Statistics version 29. As this was a cross-sectional study, the term ‘independently associated’ used throughout to describe multivariable regression findings, denotes a statistical association that persisted after adjustment for other covariates in the model, and should not be interpreted as implying a causal or temporal relationship.

### 2.7. Ethics Approval

The study was approved by the Center for Ethics in Human Research, Khon Kaen University (approval no. HE641601). Written informed consent was obtained from all participants, and confidentiality was maintained through data anonymisation.

## 3. Results

### 3.1. Patient Characteristics

Details of the participant characteristics are presented in Table 1. A total of 250 participants were enrolled between 14 January and 17 March 2023, all of whom completed the questionnaire in full and were included in the final analysis, with no post-enrolment exclusions and no missing data. Among these, 133 were male (53.2%), and 117 were female (46.8%). The mean age was 71.9 ± 9.13 years, with males being slightly younger than females (70.6 ± 9.79 vs. 73.3 ± 8.12 years). Regarding education level, 123 participants (49.2%) had completed primary education, 29 (11.6%) had completed secondary education, and 98 (39.2%) had completed tertiary education or above. The majority of participants (*n* = 226, 90.4%) were covered under the Civil Servant Medical Benefit Scheme (CSMBS), whereas 24 (9.6%) were self-paying.

Rivaroxaban was the most commonly prescribed NOAC (*n* = 80, 32.0%), followed by apixaban (*n* = 73, 29.2%), dabigatran (*n* = 62, 24.8%), and edoxaban (*n* = 35, 14.0%). The majority of participants had been taking NOACs for more than two years (*n* = 181, 72.4%), followed by one to two years (*n* = 35, 14.0%) and three to twelve months (*n* = 34, 13.6%). More than half of the participants (*n* = 158, 63.2%) were taking more than five concomitant medications. Comorbidities were present in 209 participants (83.6%), with hypertension being the most prevalent (52.4%), followed by dyslipidemia (32.0%) and diabetes mellitus (29.6%).

### 3.2. Patient Knowledge of NOAC Use

Knowledge of NOAC use was assessed using the KAQNAF-Thai in all 250 participants. The mean total score was 15.4 ± 3.04 out of a possible 23 points (67% correct responses), with a median of 15 (interquartile range [IQR] 13–18) and scores ranging from 8 to 22. Based on a cut-off of 60%, 178 participants (71.2%) were classified as having high knowledge (mean 16.9 ± 2.1; median 17, IQR 15–18; range 14–22), and 72 (28.8%) were classified as having low knowledge (mean 11.6 ± 1.3; median 12, IQR 11–13; range 8–13). The overall score range indicated that no participant scored at the absolute minimum or maximum of the scale, and scores within the high-knowledge group were not clustered near the maximum possible score, suggesting no pronounced ceiling or floor effect in this cohort. Regarding awareness of NOAC indications, 223 participants (89.2%) correctly identified the reason for their NOAC prescription, whereas 27 (10.8%) lacked awareness of their NOAC indications.

The mean correct response rate varied substantially across the six domains (Table 2). The highest rates were observed for drug–drug interactions (Domain 4, 82.5%), self-care and daily activities (Domain 6, 81.7%), and adverse drug reactions (Domain 2, 78.6%), whereas laboratory monitoring had the lowest rate (Domain 5, 36.0%). Two items were answered correctly by all participants: item 3.1 (“How often should you take this medication?”) and item 6.3 (“If you need surgery or a medical procedure, what should you do?”). Notable knowledge gaps included NOAC effectiveness after vomiting (item 1.2, 30.4%), unusual symptoms while taking a NOAC (item 2.1, 58.8%), management of a missed dose (item 3.4, 34.4%), management of accidental double dosing (item 3.6, 13.2%), and kidney and liver function monitoring requirements (items 5.1–5.2, 37.2% and 34.8%, respectively).

### 3.3. Factors Associated with Knowledge Level

Univariate analysis identified six variables meeting the *p* < 0.20 threshold for inclusion in the multivariable model (Table 3). Sex, age, education level, and awareness of NOAC indications were significantly associated with knowledge level (all *p* < 0.001). The duration of NOAC use (*p* = 0.067) and the presence of comorbidities (*p* = 0.151) also met the inclusion threshold. Concomitant medications did not meet the threshold (*p* = 0.470) and were excluded from further analysis.

In the multivariable logistic regression model, three variables were independently associated with low knowledge level (Table 4): age 75 years or older (aOR = 2.11 [95% CI 1.10–4.05], *p* = 0.025), education below the tertiary level (aOR = 17.38 [95% CI 5.03–60.01], *p* < 0.001), and lack of awareness of NOAC indications (aOR = 3.22 [95% CI 1.25–8.29], *p* = 0.016). Sex, duration of NOAC use, and presence of comorbidities were not independently associated with knowledge level (all *p* > 0.05). Model diagnostics indicated no evidence of problematic multicollinearity, with tolerance values ranging from 0.749 to 0.968 and VIF values ranging from 1.033 to 1.335. No observation had a Cook’s distance greater than 1.0; the maximum leverage value was 0.120, and the maximum absolute DFBETA among the predictor coefficients was 0.370. The model demonstrated good discrimination, with an AUC of 0.827 (95% CI 0.775–0.879), and adequate calibration, as confirmed by the Hosmer–Lemeshow goodness-of-fit test (*p* = 0.886) and by comparison of predicted probabilities with observed outcomes across deciles of predicted probability. With 72 low-knowledge events and six parameters included in the final model, the number of events per model parameter was 12.0.

## 4. Discussion

This study demonstrated that although the majority of Thai AF patients receiving NOACs met the threshold for high knowledge, clinically important gaps persisted even within this group, particularly regarding laboratory monitoring and dose management. This indicates that meeting a knowledge threshold does not equate to comprehensive treatment understanding. Older age, lower educational attainment, and lack of awareness of NOAC indications emerged as key patient-level factors independently associated with low knowledge, pointing to specific, actionable targets for educational intervention. To our knowledge, this is among the first studies to specifically characterise NOAC knowledge and its associated factors in Thai AF patients, providing evidence that can directly inform targeted educational strategies for this and similar populations.

The overall knowledge level observed in the present study is broadly comparable to findings from other countries that specifically assessed NOAC knowledge in AF patients. Using the modified JAKQ, Badak et al. reported a mean NOAC knowledge score of 73.0% among Turkish AF patients recently initiated on NOAC therapy [17], and Konieczyńska et al. similarly reported a mean JAKQ score of 60.7% among Polish AF patients on NOACs [26]. Using the Anticoagulation Knowledge Tool, Obamiro et al. found a mean knowledge score of 66% among Australian patients taking DOACs [18]. Although direct comparisons across studies are limited by differences in instruments, study populations, and cut-off criteria, these findings collectively suggest that NOAC knowledge in AF patients remains suboptimal across diverse settings. This is further supported by the systematic review by Salmasi et al., which identified significant and clinically important knowledge gaps across 21 studies of AF patients receiving oral anticoagulants, the majority of whom were taking VKAs, suggesting that knowledge deficits are a consistent concern regardless of anticoagulant type [19]. Notably, the proportion of participants with low knowledge in the present study (28.8%) was also comparable to the 29.0% with poor knowledge reported by Viola et al. in a European population receiving both VKAs and NOACs [27].

Analysis of domain-level correct response rates revealed substantial variation in knowledge across clinical areas. The highest correct response rates were observed for drug–drug interactions (Domain 4, 82.5%), self-care and daily activities (Domain 6, 81.7%), and adverse drug reactions (Domain 2, 78.6%). In contrast, laboratory monitoring was the domain with the lowest correct response rate, with a mean correct response rate of 36.0%, reflecting limited patient awareness of the recommended frequency of kidney and liver function monitoring. This finding aligns with Hernández Madrid et al., who reported that knowledge regarding the need for kidney function testing in NOAC patients was low across European countries, ranging between 15% and 38% [13]. Notably, current guidance recommends that monitoring intervals be individualised according to baseline renal function, age, and frailty, rather than following a single fixed schedule, underscoring the importance of patient understanding that such reassessment needs may vary over time and should be guided by their healthcare provider [16].

Of particular concern were knowledge gaps related to dose management and administration, spanning items from both Domain 1 and Domain 3. Correct response rates were notably low for the effectiveness of the medication after vomiting within five minutes (item 1.2, 30.4%), management of a missed dose (item 3.4, 34.4%), and management of accidental double dosing (item 3.6, 13.2%). These findings have direct clinical implications. Regarding missed doses, even a single omission may result in subtherapeutic anticoagulation due to the short half-lives of NOACs [8,9], and similar knowledge gaps in this area have been consistently identified across multiple countries and settings [19,20,28]. Accidental double dosing carries a significant bleeding risk, whereas vomiting shortly after ingestion may compromise drug absorption; in both cases, current guidance provides specific management recommendations to maintain safe and effective anticoagulation [16]. Collectively, these three gaps highlight specific and actionable targets for patient education, particularly in counselling patients on what to do when their usual medication routine is disrupted.

Two items (3.1 and 6.3) were answered correctly by all participants in the present study. This finding is consistent with the high item-difficulty indices—indicating a high proportion of correct responses—observed in an independent validation sample (0.87 and 1.00, respectively) [24], as well as with the performance on the closely related item 6.2, which was answered correctly by 98.4% of participants in the present study. Because item 6.3 was self-administered and therefore not susceptible to interviewer-related bias, its perfect response rate likely reflects near-universal knowledge of this content area. Item 3.1 was administered verbally using a standardised, verbatim procedure without prompting or confirmatory feedback (see Section 2.4). Its uniformly correct responses may instead be explained by the long treatment duration in our sample—72.4% of participants had received NOACs for more than two years—and the routine reinforcement of dosing information at each dispensing encounter. Nevertheless, a residual influence of the mode of administration cannot be excluded. Both items exhibited ceiling effects in the present study and consequently provided no discriminative information for the total score. However, as both items assess fundamental, safety-critical knowledge—appropriate medication timing and the need to disclose NOAC use before invasive procedures—we do not consider this ceiling effect to diminish their clinical importance or their retention in the instrument; rather, it may reflect appropriately high awareness of these essential safety behaviours in this population.

Regarding factors independently associated with low knowledge level, older age (75 years or older) was one of three variables independently associated in the final model (aOR = 2.11 [95% CI 1.10–4.05], *p* = 0.025). This is consistent with findings across multiple settings, where advancing age has been consistently and negatively correlated with anticoagulation knowledge, in both NOAC-specific studies [18,28] and studies assessing OAC knowledge more broadly [11,14,19]. However, as age itself is not a direct causal mechanism, this association may be at least partly explained by unmeasured factors that covary with age, including cognitive impairment, lower health literacy among older generations with historically less access to formal education, greater reliance on caregivers for medication management, and declining functional status (e.g., vision or hearing impairment) that may limit engagement with health education. As these variables were not assessed in the present study, we are unable to determine their relative contribution to the observed age association. Nonetheless, elderly patients with AF represent a particularly at-risk group, as they simultaneously carry the highest stroke risk and the lowest knowledge levels, highlighting the urgent need for targeted educational interventions in this population, regardless of the precise underlying mechanism.

Education below the tertiary level was the strongest independent predictor of low knowledge (aOR = 17.38 [95% CI 5.03–60.01], *p* < 0.001). This finding is consistent with those from multiple populations, where higher educational attainment has been consistently associated with better anticoagulation knowledge, in both NOAC-specific and broader OAC studies [13,14]. A higher education level generally correlates with better health literacy, enabling patients to comprehend complex medical information and engage more actively with their treatment [14]. The markedly high odds ratio observed in the present study may reflect the substantial variation in health literacy by educational background in Thailand, underscoring the need for tailored educational approaches for patients with lower educational attainment.

Lack of awareness of NOAC indications was one of the independent predictors of low knowledge (aOR = 3.22 [95% CI 1.25–8.29], *p* = 0.016), with 10.8% of participants unable to correctly identify the reason for their NOAC prescription. This is consistent with findings from both NOAC-specific and broader OAC studies, where patients who were unaware of their treatment indications consistently demonstrated lower overall anticoagulation knowledge [19,28]. These findings suggest that understanding the rationale for treatment is a fundamental prerequisite for broader medication knowledge. Clear communication of the indications for NOAC therapy at initiation and reinforcement at every follow-up visit is therefore warranted, as recommended by the EHRA practical guide on NOAC use [16].

This study has several noteworthy strengths. To our knowledge, it is among the first to assess NOAC knowledge and identify its associated factors in Thai AF patients using the KAQNAF-Thai, a validated Thai-language instrument, filling a previously unaddressed gap in the anticoagulation knowledge research in Thailand. The study was conducted at the Queen Sirikit Heart Center of the Northeast, the largest cardiac referral centre in northeastern Thailand, providing valuable data from a high-volume clinical setting; however, as 90.4% of participants were covered under the Civil Servant Medical Benefit Scheme, our sample may not be broadly representative of the general AF population in this region, and this is discussed further in the limitations. Additionally, this study represents the first application of the KAQNAF-Thai in clinical practice, demonstrating its feasibility for assessing NOAC knowledge in Thai AF outpatients.

Several aspects of this study’s design and sample should be considered when interpreting the findings. The single-centre design and convenience sampling of outpatients may introduce selection bias, as patients attending routine follow-up may differ from the wider AF population; although the Queen Sirikit Heart Center of the Northeast is the largest cardiac referral centre in the region, this may still restrict generalisability to smaller hospitals or other regions of Thailand. As a prospective screening log was not maintained, the response rate could not be calculated. Relatedly, our eligibility criteria—requiring capability of self-administering medication and literacy in Thai, an inherent constraint of this self-administered instrument—likely excluded patients with cognitive, functional, or literacy limitations who are at particularly high risk of inadequate knowledge, potentially underestimating the true burden in the broader AF population. Furthermore, 90.4% of participants were covered under the Civil Servant Medical Benefit Scheme, a socioeconomically distinct subgroup of the Thai population; our findings may therefore not extend to patients covered under other health insurance schemes, such as the Universal Coverage Scheme, which covers the majority of the Thai population.

Several methodological considerations also warrant caution. The cross-sectional design precludes causal inference between knowledge level and clinical outcomes. The questionnaire was self-administered, which may introduce information bias, as participants may have misunderstood certain items or provided socially desirable rather than accurate responses; the three-choice response format may have further allowed participants to guess correct answers, potentially overestimating the true level of knowledge. The binary classification of knowledge level may mask clinically important within-group variability, and the categorisation of education level in particular resulted in a sparse cell in the tertiary-education group, which likely contributed to the wide confidence interval observed for this variable. In addition, item 1.1 (awareness of NOAC indications) contributed to both the total knowledge score used to derive the outcome variable and was separately entered as a candidate predictor, introducing an element of circularity that may have inflated the observed association between this variable and low knowledge; this finding should therefore be interpreted with caution. The KAQNAF-Thai has been validated specifically in this Thai AF population, and its applicability in other linguistic or cultural contexts requires further validation. Finally, several potentially important determinants of knowledge, including clinically relevant data, health literacy, cognitive status, and the frequency and quality of NOAC counselling received prior to enrolment, were not captured in this real-world outpatient setting with limited time available per patient, and may represent important sources of unaccounted variability.

## 5. Conclusions

Despite the majority of participants demonstrating high knowledge, assessment using the KAQNAF-Thai revealed an overall mean score of approximately 67%, indicating a considerable gap in NOAC knowledge. The most critical knowledge gaps were in laboratory monitoring awareness and dose management, including missed dose management, accidental double dosing, and NOAC effectiveness after vomiting; these areas should be prioritised in patient education programmes. Outpatient pharmacists are well-positioned to identify patients at greatest risk of low knowledge—particularly those aged 75 years or older, those with below tertiary education, and those lacking awareness of their NOAC indication—and to deliver targeted education accordingly.

## Figures and Tables

**Table 1:** Baseline characteristics of patients with atrial fibrillation receiving non-vitamin K antagonist oral anticoagulants (*n* = 250).

Characteristics	
Sex, *n* (%)	
Male	133 (53.2)
Female	117 (46.8)
Age (years), mean (SD)	71.9 (9.13)
Male	70.6 (9.79)
Female	73.3 (8.12)
Education level, *n* (%)	
Primary	123 (49.2)
Secondary	29 (11.6)
Tertiary	98 (39.2)
Type of payment, *n* (%)	
Civil Servant Medical Benefit Scheme (CSMBS)	226 (90.4)
Self-pay	24 (9.6)
Type of NOACs, *n* (%)	
Dabigatran	62 (24.8)
Rivaroxaban	80 (32.0)
Apixaban	73 (29.2)
Edoxaban	35 (14.0)
Duration of NOAC use, *n* (%)	
3–12 months	34 (13.6)
1–2 years	35 (14.0)
>2 years	181 (72.4)
Concomitant medications, *n* (%)	
≤5 items	92 (36.8)
>5 items	158 (63.2)
Underlying diseases, *n* (%)	
With comorbidities ^1^	209 (83.6)
Without comorbidities	41 (16.4)

^1^ Comorbidities included hypertension, dyslipidemia, diabetes mellitus, heart failure, chronic kidney disease, valvular heart disease, coronary artery disease, and other chronic conditions.

**Table 2:** Number and percentage of participants providing correct responses to each KAQNAF-Thai item by domain (*n* = 250).

	*n* (%)
Domain 1: Basic knowledge of NOACs	
1.1 Identification of the NOAC currently used	223 (89.2)
1.2 Effectiveness of the medication after vomiting within 5 min	76 (30.4)
1.3 Impact of reduced kidney function on drug accumulation	114 (45.6)
1.4 Impact of reduced liver function on drug accumulation	123 (49.2)
1.5 Carrying medication and an anticoagulant identification card at all times	130 (52.0)
1.6 Appropriate storage of the medication	190 (76.0)
	57.1 ± 21.5% ^1^
Domain 2: Adverse drug reactions	
2.1 Recognition of unusual symptoms when taking NOAC	147 (58.8)
2.2 Situations requiring urgent medical attention	246 (98.4)
	78.6 ± 28.0% ^1^
Domain 3: Medication administration	
3.1 Frequency of medication intake	250 (100)
3.2 Duration of medication use	245 (98.0)
3.3 Taking medication at the same time daily	224 (89.6)
3.4 Management of a missed dose	86 (34.4)
3.5 Effect of missed doses on efficacy	165 (66.0)
3.6 Management of accidental double dosing	33 (13.2)
3.7 Self-adjustment or discontinuation of medication	181 (72.4)
	67.7 ± 33.0% ^1^
Domain 4: Drug–drug interactions	
4.1 Safe use of analgesics (paracetamol)	154 (61.6)
4.2 Use of herbal products or supplements	231 (92.4)
4.3 Use of other medications	234 (93.6)
	82.5 ± 18.1% ^1^
Domain 5: Laboratory monitoring	
5.1 Frequency of kidney function monitoring	93 (37.2)
5.2 Frequency of liver function monitoring	87 (34.8)
	36.0 ± 1.7% ^1^
Domain 6: Self-care and daily activities	
6.1 Activities associated with increased bleeding risk	117 (46.8)
6.2 Management before dental procedures	246 (98.4)
6.3 Management before surgery or procedures	250 (100)
	81.7 ± 30.2% ^1^

^1^ Mean percentage (SD) of participants providing correct responses to each domain.

**Table 3:** Univariate analysis of factors associated with knowledge level of NOAC use among patients with atrial fibrillation (*n* = 250).

Variables	High Knowledge Level *n* (%)	Low Knowledge Level *n* (%)	*p* Value ^1^
Sex			<0.001
Male	108 (81.2)	25 (18.8)	
Female	70 (59.8)	47 (40.2)	
Age			<0.001
<75	118 (81.9)	26 (18.1)	
≥75	60 (56.6)	46 (43.4)	
Education level			<0.001
<tertiary	83 (54.6)	69 (45.4)	
≥tertiary	95 (96.9)	3 (3.1)	
Duration of NOAC use			0.067
≤2 years	55 (79.7)	14 (20.3)	
>2 years	123 (68.0)	58 (32.0)	
Presence of comorbidities			0.151
No	33 (80.5)	8 (19.5)	
Yes	145 (69.4)	64 (30.6)	
Concomitant medications			0.470
≤5 drugs	68 (73.9)	24 (26.1)	
>5 drugs	110 (69.6)	48 (30.4)	
Awareness of NOAC Indications ^2^			<0.001
No	11 (40.7)	16 (59.3)	
Yes	167 (74.9)	56 (25.1)	

^1^ All *p* values were obtained from chi-square tests, except for education level, for which Fisher’s exact test was applied. ^2^ Defined as a correct response to item 1.1 of the KAQNAF-Thai.

**Table 4:** Multivariable logistic regression analysis of factors associated with low knowledge of NOAC use among patients with atrial fibrillation (*n* = 250).

Variable	Reference	N ^1^	β (SE)	aOR (95% CI)	*p* Value
Sex (male)	Female	25/133 vs. 47/117	−0.24 (0.35)	0.79 (0.40–1.55)	0.491
Age ≥ 75 years	Age < 75 years	46/106 vs. 26/144	0.75 (0.33)	2.11 (1.10–4.05)	0.025
Duration of NOAC use >2 years	≤2 years	58/181 vs. 14/69	0.31 (0.40)	1.36 (0.63–2.96)	0.438
Education below tertiary level	Tertiary level or above	69/152 vs. 3/98	2.86 (0.63)	17.38 (5.03–60.01)	<0.001
Presence of comorbidities	No comorbidities	64/209 vs. 8/41	0.25 (0.51)	1.29 (0.48–3.47)	0.616
Lack of awareness of NOAC indications	Aware of NOAC indication	16/27 vs. 56/223	1.17 (0.48)	3.22 (1.25–8.29)	0.016

^1^ number of participants with low knowledge/total number of participants in that category; Abbreviations: aOR, adjusted odds ratio; CI, confidence interval; SE, standard error.

## Data Availability

The data presented in this study are available on reasonable request from the corresponding author. The data are not publicly available due to privacy and ethical considerations.

## Supplementary Materials

The following supporting information can be downloaded at https://www.mdpi.com/article/10.3390/healthcare14182979/s1: Supplementary S1: Knowledge Assessment Questionnaire on NOAC Use in Thai Patients with Atrial Fibrillation (KAQNAF-Thai).

## Abbreviations

The following abbreviations are used in this manuscript:

AF	Atrial fibrillation
aORs	Adjusted odds ratios
CSMBS	Civil Servant Medical Benefit Scheme
EHRA	European Heart Rhythm Association
EPV	Events per variable
KAQNAF-Thai	Knowledge Assessment Questionnaire on NOAC Use in Thai Patients with Atrial Fibrillation
NOACs	Non-Vitamin K Antagonist Oral Anticoagulants
VKA	Vitamin K antagonist

## References

[1] Cheng S., He J., Han Y., Han S., Li P., Liao H., Guo J. (2024). Global burden of atrial fibrillation/atrial flutter and its attributable risk factors from 1990 to 2021. Europace.

[2] Joglar J.A., Chung M.K., Armbruster A.L., Benjamin E.J., Chyou J.Y., Cronin E.M., Deswal A., Eckhardt L.L., Goldberger Z.D., Gopinathannair R. (2024). 2023 ACC/AHA/ACCP/HRS Guideline for the Diagnosis and Management of Atrial Fibrillation: A Report of the American College of Cardiology/American Heart Association Joint Committee on Clinical Practice Guidelines. Circulation.

[3] Phrommintikul A., Detnuntarat P., Prasertwitayakij N., Wongcharoen W. (2016). Prevalence of atrial fibrillation in Thai elderly. J. Geriatr. Cardiol..

[4] Vinciguerra M., Dobrev D., Nattel S. (2024). Atrial fibrillation: Pathophysiology, genetic and epigenetic mechanisms. Lancet Reg. Health Eur..

[5] Hindricks G., Potpara T., Dagres N., Arbelo E., Bax J.J., Blomstrom-Lundqvist C., Boriani G., Castella M., Dan G.A., Dilaveris P.E. (2021). 2020 ESC Guidelines for the diagnosis and management of atrial fibrillation developed in collaboration with the European Association for Cardio-Thoracic Surgery (EACTS): The Task Force for the diagnosis and management of atrial fibrillation of the European Society of Cardiology (ESC) Developed with the special contribution of the European Heart Rhythm Association (EHRA) of the ESC. Eur. Heart J..

[6] Yeh C.H., Hogg K., Weitz J.I. (2015). Overview of the new oral anticoagulants: Opportunities and challenges. Arter. Thromb. Vasc. Biol..

[7] Van Gelder I.C., Rienstra M., Bunting K.V., Casado-Arroyo R., Caso V., Crijns H., De Potter T.J.R., Dwight J., Guasti L., Hanke T. (2024). 2024 ESC Guidelines for the management of atrial fibrillation developed in collaboration with the European Association for Cardio-Thoracic Surgery (EACTS). Eur. Heart J..

[8] Komen J.J., Heerdink E.R., Klungel O.H., Mantel-Teeuwisse A.K., Forslund T., Wettermark B., Hjemdahl P. (2021). Long-term persistence and adherence with non-vitamin K oral anticoagulants in patients with atrial fibrillation and their associations with stroke risk. Eur. Heart J. Cardiovasc. Pharmacother..

[9] Vitolo M., Lane D.A., Boriani G., Lip G.Y.H. (2021). The importance of adherence and persistence with oral anticoagulation treatment in patients with atrial fibrillation. Eur. Heart J. Cardiovasc. Pharmacother..

[10] Khan T.I., Kamali F., Kesteven P., Avery P., Wynne H. (2004). The value of education and self-monitoring in the management of warfarin therapy in older patients with unstable control of anticoagulation. Br. J. Haematol..

[11] Tang E.O., Lai C.S., Lee K.K., Wong R.S., Cheng G., Chan T.Y. (2003). Relationship between patients’ warfarin knowledge and anticoagulation control. Ann. Pharmacother..

[12] Wang Y., Kong M.C., Lee L.H., Ng H.J., Ko Y. (2014). Knowledge, satisfaction, and concerns regarding warfarin therapy and their association with warfarin adherence and anticoagulation control. Thromb. Res..

[13] Hernandez Madrid A., Potpara T.S., Dagres N., Chen J., Larsen T.B., Estner H., Todd D., Bongiorni M.G., Sciaraffia E., Proclemer A. (2016). Differences in attitude, education, and knowledge about oral anticoagulation therapy among patients with atrial fibrillation in Europe: Result of a self-assessment patient survey conducted by the European Heart Rhythm Association. Europace.

[14] Li C., Meng Y., Meng X., Song Y. (2023). Knowledge, attitude and practice toward oral anticoagulants among patients with atrial fibrillation. Front. Cardiovasc. Med..

[15] Rolls C.A., Obamiro K.O., Chalmers L., Bereznicki L.R.E. (2017). The relationship between knowledge, health literacy, and adherence among patients taking oral anticoagulants for stroke thromboprophylaxis in atrial fibrillation. Cardiovasc. Ther..

[16] Steffel J., Collins R., Antz M., Cornu P., Desteghe L., Haeusler K.G., Oldgren J., Reinecke H., Roldan-Schilling V., Rowell N. (2021). 2021 European Heart Rhythm Association Practical Guide on the Use of Non-Vitamin K Antagonist Oral Anticoagulants in Patients with Atrial Fibrillation. Europace.

[17] Badak O., Demir A.R., Onal T., Akgun T., Yontar O.C., Satiroglu O., Duman H., Okuyan E., Melek M., Dural I.E. (2022). Assessment of treatment patterns and patient awareness in atrial fibrillation patients using non-vitamin K antagonist oral anticoagulants (ASPECT-NOAC). Int. J. Cardiol. Heart Vasc..

[18] Obamiro K.O., Chalmers L., Lee K., Bereznicki B.J., Bereznicki L.R.E. (2018). Anticoagulation knowledge in patients with atrial fibrillation: An Australian survey. Int. J. Clin. Pract..

[19] Salmasi S., De Vera M.A., Barry A., Bansback N., Harrison M., Lynd L.D., Loewen P.S. (2019). Assessment of Condition and Medication Knowledge Gaps Among Atrial Fibrillation Patients: A Systematic Review and Meta-analysis. Ann. Pharmacother..

[20] Metaxas C., Albert V., Habegger S., Messerli M., Hersberger K.E., Arnet I. (2020). Patient Knowledge about Oral Anticoagulation Therapy Assessed during an Intermediate Medication Review in Swiss Community Pharmacies. Pharmacy.

[21] Loharattanakong P., Ritthiboon P., Hongrinya Y., Chaichun M., Taksinachanekij S., Uchaipichat V. (2016). Warfarin using knowledge and international normalized ratio goal control in outpatients of Queen Sirikit heart center of the northeast. Srinagarind Med. J..

[22] Udomdachanut P., Wongruttanachai A., Sakunrag I. (2020). Development of the Warfarin Knowledge Test in Patients Receiving Warfarin at Pathumthani Hospital. Srinagarind Med. J..

[23] Wangnirattisai N., Supakul S., Arunmanakul P. (2018). Effects of pharmaceutical care in patients receiving warfarin from the warfarin clinic at Sawanpracharak Hospital. Thai J. Pharm. Pract..

[24] Khusakul P., Inthuwong R., Pratummet P., Yindeesuk T., Nikonpitakkul K., Chaichalermpong W., Jantharuechai S., Wongvipaporn C., Uchaipichat V. (2024). Development and Validation of the Knowledge Assessment Tool for New Oral Anticoagulant Use in Patients with Atrial Fibrillation. KKU Res. J..

[25] Peduzzi P., Concato J., Kemper E., Holford T.R., Feinstein A.R. (1996). A simulation study of the number of events per variable in logistic regression analysis. J. Clin. Epidemiol..

[26] Konieczynska M., Sobieraj E., Bryk A.H., Debski M., Polak M., Podolec P., Malecka B., Pajak A., Desteghe L., Heidbuchel H. (2018). Differences in knowledge among patients with atrial fibrillation receiving non-vitamin K antagonist oral anticoagulants and vitamin K antagonists. Kardiol. Pol..

[27] Viola R., Fekete H., Csoka I. (2017). Patients’ knowledge on oral anticoagulant treatment in Hungary. Int. J. Clin. Pharm..

[28] Desteghe L., Engelhard L., Raymaekers Z., Kluts K., Vijgen J., Dilling-Boer D., Koopman P., Schurmans J., Dendale P., Heidbuchel H. (2016). Knowledge gaps in patients with atrial fibrillation revealed by a new validated knowledge questionnaire. Int. J. Cardiol..

