# Supplementary material for: Knowledge of Non-Vitamin K Antagonist Oral Anticoagulants and Associated Factors Among Patients with Atrial Fibrillation: A Cross-Sectional Study in Thailand
Source: Healthcare (Basel). 2026 Sep 12;14(18):2979. doi: 10.3390/healthcare14182979 (PMC13606563; doi:10.3390/healthcare14182979)
Supplement: Supplementary file 1 [file healthcare-14-02979-s001.zip › healthcare-4509371-supplementary.pdf]

Supplementary S1. Knowledge Assessment Questionnaire on NOAC Use in Thai Patients with Atrial Fibrillation (KAQNAF-Thai)

Instructions

Please select the most appropriate answer for each question. If you are unsure, please select "Not sure."

Domain 1: Basic knowledge of NOACs

1.1 Which oral anticoagulant (NOAC) are you currently taking?
- (Researcher verification using patient medication)

1.2 If vomiting occurs within 5 minutes after taking the medication, the drug will still be effective.
- True
- False
- Not sure

1.3 Reduced kidney function may lead to increased accumulation of this medication in the body.
- True
- False
- Not sure

1.4 Reduced liver function may lead to increased accumulation of this medication in the body.
- True
- False
- Not sure

1.5 You should always carry your medication and anticoagulant identification card with you.
- True
- False
- Not sure

1.6 How should this medication be stored?
- Keep the medication in a refrigerator
- Keep the medication away from sunlight, heat, and moisture
- Remove tablets from the blister pack and store them in a pillbox

Domain 2: Adverse drug reactions

2.1 What unusual symptoms may occur while taking this medication?
- Weakness of the arms or legs, facial drooping, or drooping of the mouth
- Unusual bleeding or bruising
- All of the above

2.2 Which of the following situations requires urgent medical attention?
- Minor bruising

- Bleeding that does not stop or excessive bleeding
- Bleeding from the gums

Domain 3: Medication administration

3.1 How often should you take this medication?
- Once daily
- Twice daily (every 12 hours)
- Not sure

3.2 How long should you continue taking this medication?
- 3 months
- 6 months
- Lifelong

3.3 This medication should be taken at the same time every day.
- True
- False
- Not sure

3.4 If you miss a dose, you should take an extra dose the next time.
- True
- False
- Not sure

3.5 Missing a dose may reduce the effectiveness of the medication.
- True
- False
- Not sure

3.6 If you accidentally take an extra dose, what should you do?
- Do nothing
- Monitor for abnormal symptoms
- Go to the hospital immediately

3.7 If your symptoms improve, you can stop or reduce the medication by yourself.
- True
- False
- Not sure

Domain 4: Drug–drug interactions

4.1 Paracetamol is a safe medicine for relieving pain and reducing fever.
- True
- False
- Not sure

4.2 If you want to take herbal products or vitamins, what should you do?
- Take them immediately
- Do not take them
- Consult a pharmacist and inform them that you are taking an anticoagulant

4.3 If you need to take other medications, what should you do?
- Take them immediately
- Do not take them
- Consult a pharmacist and inform them that you are taking an anticoagulant

Domain 5: Laboratory monitoring
5.1 Patients taking this medication should have their kidney function checked at least once a year.
- True
- False
- Not sure

5.2 Patients taking this medication should have their liver function checked at least once a year.
- True
- False
- Not sure

Domain 6: Self-care and daily activities
6.1 What activities may increase the risk of side effects while taking this medication?
- Normal daily activities
- Activities with risk of falling, bleeding, or injury
- Not sure

6.2 If you need a dental procedure (e.g., tooth extraction), what should you do?
- Inform the doctor/dentist that you are taking an anticoagulant
- Do not inform anyone
- Not sure

6.3 If you need surgery or a medical procedure, what should you do?
- Inform the doctor that you are taking an anticoagulant
- Do not inform anyone
- Not sure

Scoring system (for Methods / Supplementary use)
- Each correct answer = **1 point**
- Incorrect or "Not sure" = **0 points**
- Total score = **0–23 points**

Knowledge level classification:
- **High knowledge:** ≥60% (≥14 points)
- **Low knowledge:** <60% (<14 points)

Answer key

Questions	Correct answers
1.2	False
1.3	True
1.4	True
1.5	True
1.6	Keep away from sunlight, heat, moisture
2.1	All of the above
2.2	Bleeding that does not stop or excessive bleeding
3.1	The correct answer depends on the medication prescribed to the patient, and the pharmacist will determine whether the patient's response is correct.
3.2	Lifelong
3.3	True
3.4	False
3.5	True
3.6	Monitor for abnormal symptoms
3.7	False
4.1	True
4.2	Consult pharmacist
4.3	Consult pharmacist
5.1	True
5.2	True
6.1	Activities with risk of injury
6.2	Inform doctor/dentist
6.3	Inform doctor
